# Association between single nucleotide polymorphisms in *EPAS1* and *PPARA* genes and high altitude polycythemia in Chinese Tibetan population

**DOI:** 10.3389/fgene.2025.1519108

**Published:** 2025-03-06

**Authors:** Ziyi Chen, Zhaomei Dong, Rong Zeng, Mengna Xu, Yuanyuan Zhang, Qu Dan, Guangming Wang

**Affiliations:** ^1^ School of Clinical Medicine, Dali University, Dali, Yunnan, China; ^2^ Department of Reproductive Medicine, First Affiliated Hospital of Dali University, Dali, Yunnan, China; ^3^ Department of Geriatrics, The Second People’s Hospital of Kunming, Kunming, Yunnan, China; ^4^ Department of Infection, Puer People’s Hospital, Puer, Yunnan, China; ^5^ Medicine Department, School of Clinical Medicine, Dali University, Dali, Yunnan, China; ^6^ Department of Laboratory, Tibet Autonomous Region People’s Hospital, Lhasa, Tibet, China; ^7^ Center of Genetic Testing, The First Affiliated Hospital of Dali University, Dali, Yunnan, China

**Keywords:** *EPAS1*, *PPARA*, high altitude polycythemia, single nucleotide polymorphisms, Tibetans

## Abstract

**Background:**

High altitude polycythemia (HAPC) is a disease with high morbidity and great harm in high altitude populations. It has been shown that Single Nucleotide Polymorphisms (SNPs) correlate with the genetic basis of adaptation to plateau hypoxia in Tibetan populations. The *EPAS1* and *PPARA* genes are involved in hypoxia adaptation by encoding transcription factors in Tibetan populations at high altitude. The aim of this study was to investigate the association of *EPAS1* and *PPARA* gene locus polymorphisms with genetic susceptibility to HAPC in the Chinese Tibetan population.

**Methods:**

We included 78 HAPC patients and 84 healthy controls, and genotyped the *EPAS1* gene SNP loci (rs6735530, rs6756667, rs7583392, and rs12467821) and *PPARA* rs6520015 by using TaqMan polymerase chain reaction. Logistic regression was used to analyze the association between these SNPs and HAPC; interactions between SNPs were also predicted by multifactorial dimensionality reduction (MDR) analysis.

**Results:**

We found that the *PPARA* rs6520015 polymorphism was not associated with the risk of HAPC in the Chinese Tibetan population; *EPAS1* rs6735530, rs6756667, rs7583392, and rs12467821 increased the risk of HAPC in some models. Haplotype TCAGC decreases the risk of HAPC; Haplotype TTGAT increases the risk of HAPC; and *EPAS1* rs7583392 is in complete linkage disequilibrium with rs12467821. The best prediction model was the *EPAS1* rs6756667 unit point model, but the P value was greater than 0.05 in all three models, which was not statistically significant.

**Conclusion:**

The present findings suggest that among the Tibetan population in China, There is an association between *EPAS1* rs6735530, rs6756667, rs7583392, and rs12467821 and the risk of HAPC, and that there is no significant correlation between *PPARA* rs6520015 and the risk of HAPC.

## 1 Introduction

High altitude polycythemia (HAPC) is a chronic plateau disease in which the organism lives for a long period of time on a plateau at an altitude of more than 2,500 m above sea level, and the lack of oxygen in the tissues leads to the compensatory overproliferation of erythrocytes (women’s hemoglobin ≥19 g/dL, men’s hemoglobin ≥ 21 g/dL), increased blood viscosity, and clinical symptoms such as dizziness, shortness of breath, and chest tightness (León-Velarde et al., 2005; Beall and Goldstein, 1987; Wu, 2005). HAPC is the most prevalent and harmful chronic high altitude disease among high altitude residents. It is estimated that up to 5%–10% of high altitude residents may develop HAPC(1). There are differences in the prevalence of HAPC by race, gender, age, altitude of residence, and duration of residence (Meyer et al., 2017). The risk of HAPC is associated with genetic, environmental and physiological factors. In the low oxygen environment of plateau, lowlanders are more likely to suffer from HAPC than highlanders (Garrido et al., 1996). It has been reported that the prevalence of HAPC is lower in Tibetan long-established populations at the same altitude compared to migrated Han Chinese populations (Wu et al., 2005). Genome-wide association studies (GWAS) have confirmed that the differences in genetic background between lowlanders and highlanders are the result of natural selection of genomic loci over hundreds of generations in highlanders. SNPs in genes such as *EPAS1*, *PPARA*, *EGLN1*, *EDNRA*, and *PTEN* were found to correlate with hemoglobin (Hb) concentrations in high altitude Tibetan populations, and they found that most of these genes play a role in the HIF-1 pathway (Simonson et al., 2010). In addition, Mallik N suggests that gain-of-function mutations in *EPAS1* and loss-of-function mutations in *EGLN1* lead to constitutive activation of EPO signaling and thus to HAPC. Strong and significant associations between Hb concentrations and haplotypic variation in *PPARA* and *EGLN1* provide evidence of a genetic mechanism for the form of high-altitude acclimation that characterizes Tibetan populations (Simonson et al., 2010; Mallik et al., 2021). Polymorphisms in the *CYP17A1* and *CYP2E1* gene loci have been shown to play a role in mediating susceptibility to HAPC (Xu et al., 2015a).

Endothelial-type PAS structural domain protein 1 (*EPAS1*), also known as hypoxia-inducible factor 2 (HIF-2ɑ), is encoded by the *EPAS1* gene located at 2p21, and is a protein composed of 870 amino acids (Karaghiannis et al., 2023). Numerous studies have shown that polymorphisms in *EPAS1* are associated with the pathogenesis, pathologic staging, progression, and prognosis of cancers such as colorectal cancer, hepatocellular carcinoma, esophageal squamous cell carcinoma, non-small cell lung cancer, and papillary thyroid carcinoma (Baba et al., 2010; Mohammed et al., 2011; Bangoura et al., 2004; Islam et al., 2020; Zhen et al., 2021; Zhang et al., 2023). *EPAS1* rs7583392, rs6756667, and rs12467821 have been reported to be closely associated with plateau diseases and are thought to be related to high altitude acclimatization in Tibetans (Li et al., 2024; Basang et al., 2015; Bhandari et al., 2017; Pan et al., 2019). The hypoxia-inducible factor (HIF) oxygen signaling pathway plays an important role in hypoxia acclimatization in Tibetan populations (Simonson et al., 2010; Wang et al., 2011; Beall et al., 2010; Peng et al., 2011), but whether there is an association with HAPC is not yet known. The protein stability of HIF-2ɑ increases when the body is in a hypoxic state, and accumulated HIF-2ɑ continues to stimulate the HIF pathway, promoting transcription of downstream genes involved in erythropoiesis, hypoxic metabolism, inflammation, angiogenesis, and tumorigenesis (Wang et al., 2023; van Patot and Gassmann, 2011; Davis et al., 2022).

The peroxisome proliferator-activated receptor α gene (*PPARA*) is located on chromosome 22 and encodes peroxisome proliferator-activated receptor α (*PPARα*), a member of the nuclear receptor family of transcription factors, which regulates physiological processes, such as lipid metabolism, gluconeogenesis, and immune responses, particularly fatty acid β-oxidation in mitochondria and peroxisomes (Standage et al., 2017; Jay and Ren, 2007). Polymorphisms in the *PPARA* have been reported to be associated with the risk of liver disease, celiac disease, type 2 diabetes, and hyperlipidemia, among others (Jay and Ren, 2007; Li et al., 2014; Mostowy et al., 2016; Eurlings et al., 2002). A variant in the “C” allele of *PPARA* rs6520015 affects cardiac ejection function after acute plateau exposure (Standage et al., 2017; Yang et al., 2019). The *PPARA* promoter is associated with vascular function, target gene transcription and hemoglobin concentration during hypoxia in a Tibetan population (Shen et al., 2020).

In Tibetan populations at high altitude, *EPAS1* and *PPARA* genes are involved in hypoxia adaptation by encoding transcription factors, and they play important roles in hypoxia-responsive pathways and oxidative stress (Simonson et al., 2010). However, whether the polymorphism of these genes is related to the incidence rate of HAPC in Chinese Tibetan population still needs to be investigated. Therefore, the aim of this study was to investigate the genetic relationship between polymorphisms in the *EPAS1* and *PPARA* loci and HAPC in the Chinese Tibetan population.

## 2 Materials and methods

This study was approved by the Research Ethics Committee of the First Affiliated Hospital of Dali University (approval number: DFY20171210002, date: 10 December 2017). We selected 162 participants from the Tibetan population in the Lhasa area of Tibet, all of whom were residents of high altitude areas. Among them, 78 cases were HAPC patients in the experimental group and 84 cases were healthy people in the control group. The inclusion criteria of the experimental group were: (León-Velarde et al., 2005): Hb ≥21 g/dL for men and ≥19 g/dL for women; (Beall and Goldstein, 1987); long-term residence in the plateau area with an elevation of more than 3000 m above sea level; and (Wu, 2005) having three or more of the following symptoms: headache, dizziness, fatigue, cyanosis, sleep disturbance, conjunctival congestion, and purplish skin. Patients with true cytosis and other secondary erythrocytosis; cardiovascular and cerebrovascular diseases, autoimmune diseases, malignant tumors, immune system diseases, and neurological diseases; and incomplete relevant data were excluded from the study. Clinical data: red blood cell count, hemoglobin, and hematocrit were also collected from the 2 groups.

### 2.1 DNA extraction and quality control

**FIGURE 1:**
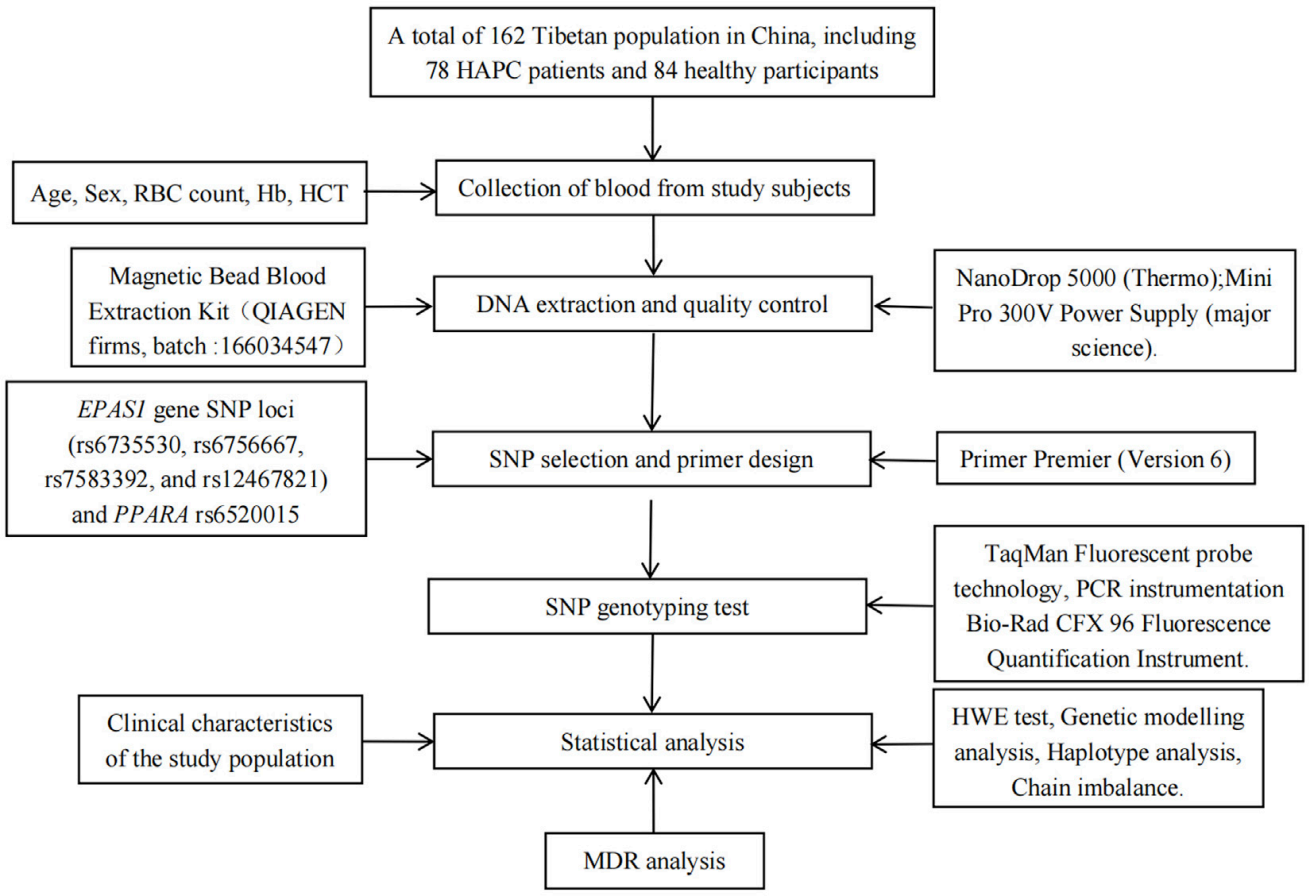
Flow chart of this study.

The flow chart of this study is shown in Figure 1. Fasting venous blood (2 mL) was taken into EDTA tubes, and the collected blood samples were stored in −20°C refrigerator for subsequent use, genomic DNA was extracted using DNA extraction kit, the integrity of genomic DNA was detected by agarose gel electrophoresis, and DNA concentration was detected by microspectrophotometer.

### 2.2 Selection and analysis of gene loci

The National Center for Biotechnology Information (NCBI, USA, http://www.ncbi.nlm.nih.gov/) database was used to obtain reference information of SNPs of *EPAS1* and *PPARA* genes. *PPARA* rs6520015 (C/T) and four loci of the *EPAS1* gene, rs6735530 (C/T), rs6756667 (A/G), rs7583392 (A/G), and rs12467821 (C/T), were selected.

### 2.3 Genotyping

TaqMan fluorescent probe technology was used for genotyping detection of polymorphic loci, and different fluorescence signals were detected by real-time fluorescence quantitative PCR. The reaction conditions were as follows: pre-denaturation at 95°C for 5 min, 40 cycles (denaturation at 95°C for 10 s, annealing at 60°C for 30 s, and extension at 72°C for 2 min), and finally extension at 16°C for 5 min. The primer probe sequences for the five SNP loci (rs6520015, rs6735530, rs6756667, rs7583392, and rs12467821) are shown in Table 1.

**TABLE 1:** Primer probe sequences.

SNP ID	Polymorphism	Probes	Sequences
rs6520015	C/T	CC genotype	5′-AGT​GGC​ATC​TTA​TCC​AGG​AGC​CAC​C-3′
		TT genotype	5′-AGT​GGC​ATC​TTA​TCC​AGG​AGC​CAC​T-3′
		reverse primer	5′-AAT​AAA​CTT​GAG​ATT​GCA​AGT​TTT​C-3′
rs6735530	C/T	CC genotype	5′-CAC​TGC​TCC​TCA​TTC​ATC​ATA​TGC​C-3′
		TT genotype	5′-CAC​TGC​TCC​TCA​TTC​ATC​ATA​TGC​T-3′
		reverse primer	5′-TGG​GCT​TGG​CAG​AAC​TAA​TAA​AGA​C-3′
rs6756667	A/G	AA genotype	5′-CAA​GAG​TTG​ATG​CTG​GAT​TGT​GGC​A-3′
		GG genotype	5′-CAA​GAG​TTG​ATG​CTG​GAT​TGT​GGC​G-3′
		reverse primer	5′-AGA​CAG​ATA​AAC​TGC​TGT​AAG​GTG​A-3′
rs7583392	A/G	AA genotype	5′-GCC​TCA​TAT​ATA​CCA​CCC​CAC​TCA​A-3′
		GG genotype	5′-GCC​TCA​TAT​ATA​CCA​CCC​CAC​TCA​G-3′
		reverse primer	5′-CCT​GTT​TTA​TTA​AAT​ATT​ATT​GCA​C-3′
rs12467821	C/T	CC genotype	5′-TGT​ATA​CTT​CAA​ATG​GGT​GTG​TGT​C-3′
		TT genotype	5′-TGT​ATA​CTT​CAA​ATG​GGT​GTG​TGT​T-3′
		reverse primer	5′-TTG​GGG​TCA​AAT​TTA​CCT​TCA​ATA​A-3′

SNP, single nucleotide polymorphism.

### 2.4 Statistical analysis

SPSS 27.0 software was used for statistical analysis (*P* < 0.05 is statistically different), and t-test and chi-square test were used for numerical and categorical data, respectively. Logistic regression was used to assess the association between genotyping of polymorphisms of *EPAS1* and *PPARA* gene locus and the risk of occurrence of HAPC in the present study by constructing multiple genetic models (allele, dominant model, recessive model, codominant model, overdominant model). The HWE (Hardy-Weinberg equilibrium) test was used to assess the population genetics of the control group, and if *P* > 0.05, it indicated that the data of this study followed the law of population genetics and was representative of the population.

### 2.5 Other analyses

Linkage disequilibrium determination and haplotype analysis were performed using online SHEsis (http://analysis.bio-x.cn/myAnalysis.php) to analyze the association between gene haplotypes and the risk of developing HAPC. Multi-factor dimensionality reduction software MDR3.0.2 was used to analyze the interactions among the five gene loci studied, and the Cross-validation consistency and Testing accuracy of each model were calculated, and *P* < 0.05 was statistically significant.

## 3 Result

### 3.1 Clinical characteristics of study subjects

Table 2 shows the demographic and clinical characteristics of these participants. The mean age of the control group and cases was 45.63 ± 18.208 years and 48.41 ± 14.671, respectively (*P* > 0.05). There were significant differences in gender, red blood cell count, hemoglobin, and hematocrit between the two groups (*P* < 0.05 statistically significant).

**TABLE 2:** Analysis of the basic clinical characteristics of the study population.

Characteristics	Control (n = 84)	HAPC (n = 78)	*P*
Age (year)	45.63 ± 18.208	48.41 ± 14.671	0.285
M/F	31/53	59/19	<0.01
RBC count (10^9^/L)	4.9155 ± 0.57131	7.0009 ± 0.69195	<0.01
Hb (g/L)	143.01 ± 17.422	221.49 ± 18.721	<0.01
HCT (%)	42.377 ± 6.1034	64.779 ± 5.8298	<0.01

M/F, Male/Female; Hb, Hemoglobin; HCT, hematocrit.

### 3.2 Hardy weinberg equilibrium (HWE) test analysis

The HWE-P of five SNPs (rs6520015, rs6735530, rs6756667, rs7583392, and rs12467821) in the control and experimental groups were all >0.05, indicating that the gene frequencies observed in the HAPC study population were representative of the gene distributions observed in the general population, and the results are shown in Table 3.

**TABLE 3:** HWE balance of gene loci.

SNPs	Control			HAPC		
	N	χ^2^	HWE-P	N	χ^2^	HWE-P
rs6520015	84	0.104	0.747	78	0.279	0.597
rs6735530	84	0.106	0.744	78	1.685	0.194
rs6756667	84	0.470	0.493	78	1.067	0.302
rs7583392	84	0.015	0.904	78	1.467	0.226
rs12467821	84	0.065	0.799	78	0.737	0.391

SNP, single nucleotide polymorphism.

### 3.3 Association analysis of *EPAS1* and *PPARA* gene locus polymorphisms and the risk of HAPC occurrence

To assess the association of SNPs in the *EPAS1* and *PPARA* gene with the effect of HAPC disease, we analyzed the association of rs6520015, rs6735530, rs6756667, rs7583392, and rs12467821 with the risk of developing HAPC in 162 participants by constructing multiple genetic model analyses, including codominant, dominant, recessive, and overdominant models, and by using binary logistic regression analysis. The genotyping and allele frequency distributions of the healthy and HAPC groups are summarized in Table 4. The results showed that no association was found between *PPARA* rs6520015 and the risk of HAPC disease in the Tibetan population (*P* > 0.05).

**TABLE 4:** Association analysis of gene locus polymorphisms with the risk of developing HAPC.

SNP ID	Model	Genotype	Control	Case	OR (95%CI)	*P* Value
rs6520015	Codominant	TT	49 (58.3%)	43 (55.13%)	Reference	
		CT	31 (36.9%)	31 (39.74%)	1.140 (0.598–2.171)	0.691
		CC	4 (4.8%)	4 (5.13%)	1.140 (0.269–4.834)	0.859
	Dominant	TT	49 (58.3%)	43 (55.1%)	Reference	
		CT+CC	35 (41.7%)	35 (44.9%)	1.140 (0.612–2.123)	0.681
	Recessive	TT+CT	80 (95.2%)	74 (94.9%)	Reference	
		CC	4 (4.8%)	4 (5.1%)	1.081 (0.261–4.479)	0.914
	Overdominant	TT+CC	53 (63.1%)	47 (60.26%)	Reference	
		CT	31 (36.9%)	31 (39.74%)	1.128 (0.598–2.126)	0.710
	Allele	T	129 (76.8%)	117 (75.0%)	Reference	
		C	39 (23.2%)	39 (25.0%)	1.103 (0.662–1.835)	0.707
rs6735530	Codominant	CC	36 (42.9%)	26 (33.3%)	Reference	
		CT	39 (46.4%)	33 (42.3%)	1.172 (0.591–2.324)	0.651
		TT	9 (10.7%)	19 (24.4%)	2.923 (1.142–7.482)	0.025
	Dominant	CC	36 (42.9%)	26 (33.3%)	Reference	
		CT+TT	48 (57.1%)	52 (66.7%)	1.500 (0.792–2.842)	0.214
	Recessive	CC+CT	75 (89.3%)	59 (75.6%)	Reference	
		TT	9 (10.7%)	19 (24.4%)	2.684 (1.132–6.363)	0.025
	Overdominant	CC+TT	45 (53.6%)	45 (57.7%)	Reference	
		CT	39 (46.4%)	33 (42.3%)	0.846 (0.455–1.575)	0.598
	Allele	C	111 (66.1%)	85 (54.5%)	Reference	
		T	57 (33.9%)	71 (45.5%)	1.627 (1.039–2.548)	0.034
rs6756667	Codominant	AA	65 (77.4%)	38 (48.7%)	Reference	
		AG	17 (20.2%)	30 (38.5%)	3.019 (1.474–6.183)	0.003
		GG	2 (2.4%)	10 (12.8%)	8.553 (1.779–41.112)	0.007
	Dominant	AA	65 (77.4%)	38 (48.7%)	Reference	
		AG+GG	19 (22.6%)	40 (51.3%)	3.601 (1.830–7.087)	0.000
	Recessive	AA+AG	82 (97.6%)	68 (87.2%)	Reference	
		GG	2 (2.4%)	10 (12.8%)	6.029 (1.277–28.460)	0.023
	Overdominant	AA+GG	67 (79.8%)	48 (61.5%)	Reference	
		AG	17 (20.2%)	30 (38.5%)	2.463 (1.222–4.965)	0.012
	Allele	A	147 (87.5%)	106 (67.9%)	Reference	
		G	21 (12.5%)	50 (32.1%)	3.302 (1.872–5.824)	0.000
rs7583392	Codominant	GG	61 (72.6%)	39 (50.0%)	Reference	
		AG	21 (25.0%)	29 (37.2%)	2.160 (1.083–4.309)	0.029
		AA	2 (2.4%)	10 (12.8%)	7.821 (1.626–37.608)	0.010
	Dominant	GG	61 (72.6%)	39 (50.0%)	Reference	
		AG+AA	23 (27.4%)	39 (50.0%)	2.652 (1.380–5.098)	0.003
	Recessive	GG+AG	82 (97.6%)	68 (87.2%)	Reference	
		AA	2 (2.4%)	10 (12.8%)	6.029 (1.277–28.460)	0.023
	Overdominant	GG+AA	63 (75.0%)	49 (62.8%)	Reference	
		AG	21 (25.0%)	29 (37.2%)	1.776 (0.905–3.485)	0.095
	Allele	G	143 (85.1%)	107 (68.6%)	Reference	
		A	25 (14.9%)	49 (31.4%)	2.619 (1.522–4.508)	0.001
rs12467821	Codominant	CC	62 (73.8%)	39 (50.0%)	Reference	
		CT	20 (23.8%)	30 (38.5%)	2.385 (1.192–4.770)	0.014
		TT	2 (2.4%)	9 (11.5%)	7.154 (1.468–34.859)	0.015
	Dominant	CC	62 (73.8%)	39 (50.0%)	Reference	
		CT+TT	22 (26.2%)	39 (50.0%)	2.818 (1.459–5.444)	0.002
	Recessive	CC+CT	82 (97.6%)	69 (88.5%)	Reference	
		TT	2 (2.4%)	9 (11.5%)	5.348 (1.118–25.584)	0.036
	Overdominant	CC+TT	64 (76.2%)	48 (61.5%)	Reference	
		CT	20 (23.8%)	30 (38.5%)	2.000 (1.015–3.941)	0.045
	Allele	C	144 (85.7%)	108 (69.2%)	Reference	
		T	24 (14.3%)	48 (30.8%)	2.667 (1.539–4.621)	0.000

SNP, single nucleotide polymorphism; OR, odds ratio; CI, confidence interval.

In *EPAS1* rs6735530, the genotyping distribution was as follows: 33.3% CC, 42.3% CT, and 24.4% TT in the HAPC group, while it was 42.9% CC, 46.4% CT, and 10.7% TT in the control group. Using the C allele as a reference, allele T was correlated with the risk of developing HAPC [OR = 1.627, CI = (1.039–2.548), *P* = 0.034]. Under the codominant model, TT was associated with susceptibility to HAPC compared to the CC genotype [OR = 2.923, CI = (1.142–7.482), *P* = 0.025], and under the recessive model, individuals with the TT genotype had an elevated risk of developing the disease [OR = 2.684, CI = (1.132–6.363), *P* = 0.025].

In *EPAS1* rs6756667, the genotyping distribution was as follows: 48.7% AA, 38.5% AG, and 12.8% GG in HAPC group, while it was 77.4% AA, 20.2% AG, and 2.4% GG in control group. The frequency of allele G was elevated in HAPC patients compared to allele A [OR = 3.302, CI = (1.872–5.824), *P* = 0.000]. Under the codominant model, using the AA genotype as a reference, genotypes AG and GG both seemed to raise the risk of HAPC [AG:OR = 3.019, CI = (1.474–6.183), *P* = 0.003; GG:OR = 8.553, CI = (1.779–41.112), *P* = 0.007]. Individuals carrying the G allele (AG + GG) under the dominant model {OR = 3.601, CI = [(1.830–7.087], *P* = 0.000}, the GG genotype under the recessive model [OR = 6.029, CI = (1.277–28.460), *P* = 0.023], and carrying the heterozygous AG genotype under the overdominant model [OR = 2.463, CI = (1.222–4.965), *P* = 0.012] were all more likely to have HAPC.

In *EPAS1* rs7583392, the genotyping distribution was as follows: 50.0% GG, 37.2% AG, and 12.8% AA in HAPC group; while it was 72.6% GG, 25.0% AG, and 2.4% AA in control group. rs7583392 allele G/A [A vs. G:OR = 2.619, CI = (1.522–4.508), *P* = 0.001], codominant model [AG vs. GG: OR = 2.160, CI = (1.083–4.309), *P* = 0.029; AA vs. GG:OR = 7.821, CI = (1.626–37.608), *P* = 0.010], dominant model [AG-AA vs. GG:OR = 2.652,CI = (1.380–5.098), *P* = 0.003], and recessive model [AA vs. GG-AG:OR = 6.029, CI = (1.277–28.460), *P* = 0.023] were risk factors for HAPC.

In *EPAS1* rs12467821, the genotyping distribution was as follows: 50.0% CC, 38.5% CT, and 11.5% TT in the HAPC group; while it was 73.8% CC, 23.8% CT, and 2.4% TT in the control group. The susceptibility to HAPC was found in allele C/T [T vs. C:OR = 2.667, CI = (1.539–4.621), *P* = 0.000], codominant model [CT vs. CC:OR = 2.385, CI = (1.192–4.770), *P* = 0.014; TT vs. CC:OR = 7.154, CI = (1.468–34.859), *P* = 0.015], dominant model [CT-TT vs. CC:OR = 2.818, CI = (1.459–5.444), *P* = 0.002], recessive model [TT vs. CC-CT:OR = 5.348,CI = (1.118–25.584), *P* = 0.036], and overdominant models [CT vs. CC-TT:OR = 2.000,CI = (1.015–3.941), *P* = 0.045] would be significantly higher.

### 3.4 Linkage disequilibrium of SNPs in *EPAS1* and *PPARA* genes and their haplotype association with HAPC

**FIGURE 2:**
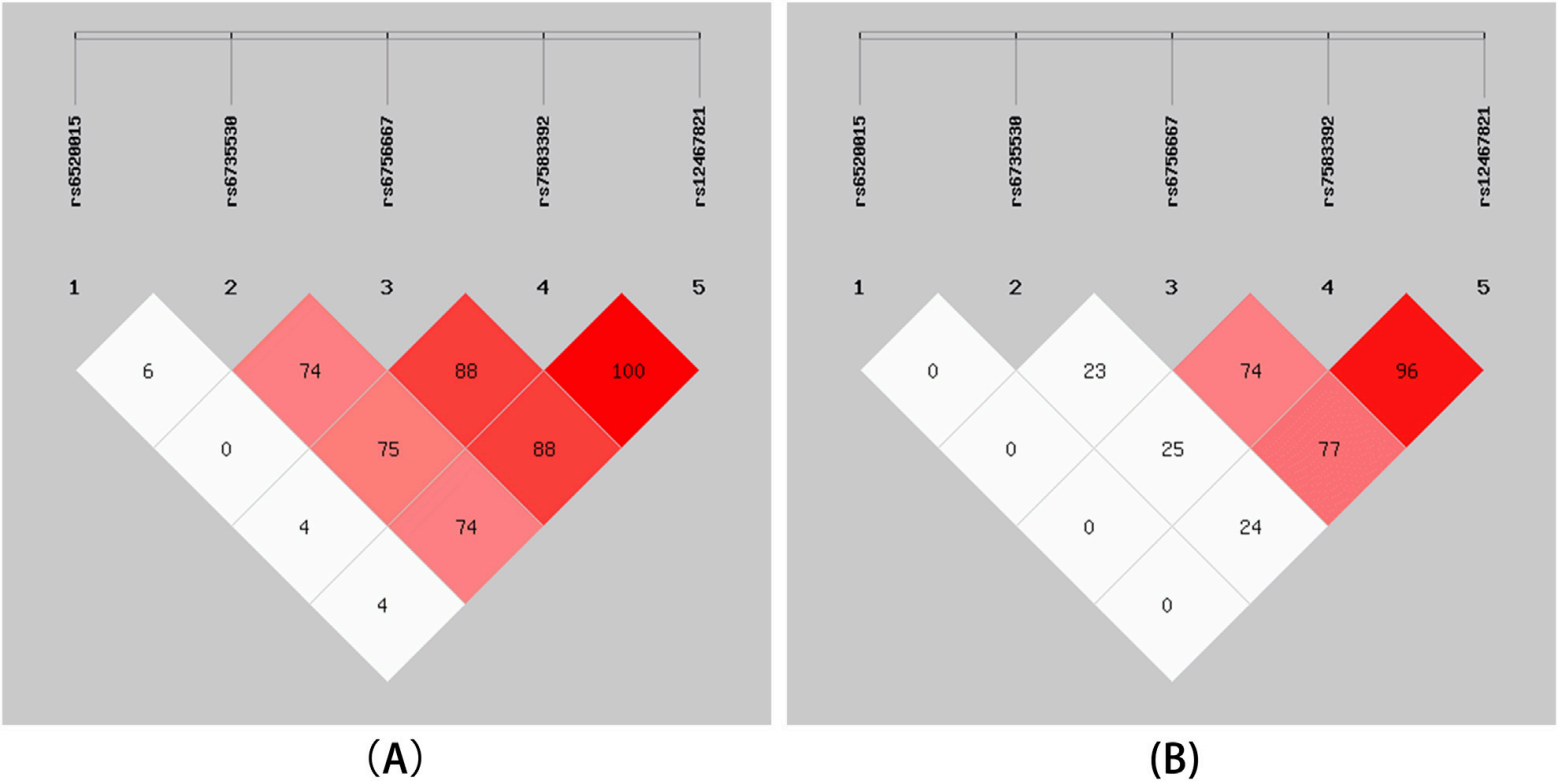
Linkage disequilibrium (LD) of 5 SNPs. **(A)** The numbers inside the diamonds indicate the D’ for pairwise analyses. **(B)** The numbers inside the diamonds indicate the r2 for pairwise analyses.

Five SNPs loci of this study were analyzed using online SHEsis. The SNP order of haolotype was rs6520015, rs6735530, rs6756667, rs7583392, and rs12467821. Based on the analysis results, haplotypes with frequencies below 3% were removed, and a total of six haplotypes were obtained. In descending order of frequency, they were TCAGC, TTGAT, TTAGC, CCAGC, CTGAT, and CTAGC; among them, two haplotypes, TCAGC and TTGAT, differed between the HAPC group and the control group (*P* < 0.05), and TCAGC appeared in HAPC cases with a significantly lower frequency than that of the control group, so the TCAGC haplotype had a protective effect against HAPC had a protective effect, while TTGAT appeared significantly more frequently in HAPC cases than in the control group, so the TTGAT haplotype increased the risk of HAPC; the specific results of the analyses are shown in Table 5. In Figure 2, it can be seen that there was a complete linkage disequilibrium between *EPAS1* rs7583392 and rs12467821 (D’ = 1.00, r2 = 0.96).

**TABLE 5:** Haplotype analysis of gene locus polymorphisms and risk of HAPC occurrence.

Haplotypes	HAPC group	Control group	χ^2^	*P* Value	OR (95%CI)
TCAGC	59.25 (38.0%)	86.45 (51.5%)	4.847	0.028	0.599 (0.379–0.947)
TTGAT	30.29 (19.4%)	12.10 (7.2%)	11.646	0.001	3.288 (1.616–6.692)
TTAGC	18.60 (11.9%)	24.64 (14.7%)	0.355	0.552	0.821 (0.429–1.572)
CCAGC	16.84 (10.8%)	20.71 (12.3%)	0.095	0.758	0.898 (0.452–1.785)
CTGAT	8.52 (5.5%)	5.57 (3.3%)	1.045	0.307	1.757 (0.589–5.239)
CTAGC	8.28 (5.3%)	9.14 (5.4%)	0.001	0.976	1.015 (0.385–2.674)

OR, odds ratio; CI, confidence interval.

### 3.5 Gene-gene interaction analysis

**FIGURE 3:**
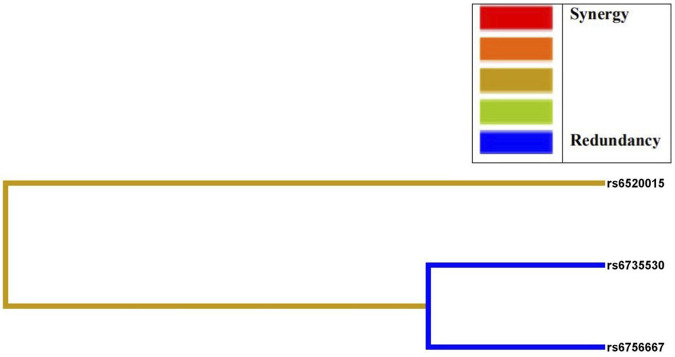
Dendrogram of SNP-SNP interactions. The blue line indicates that the SNPs have a redundancy effect in regulating the risk of HAPC, and the gold line represents the intermediate point between synergistic and redundancy effects. The closer the loci are, the stronger the interactions are.

**FIGURE 4:**
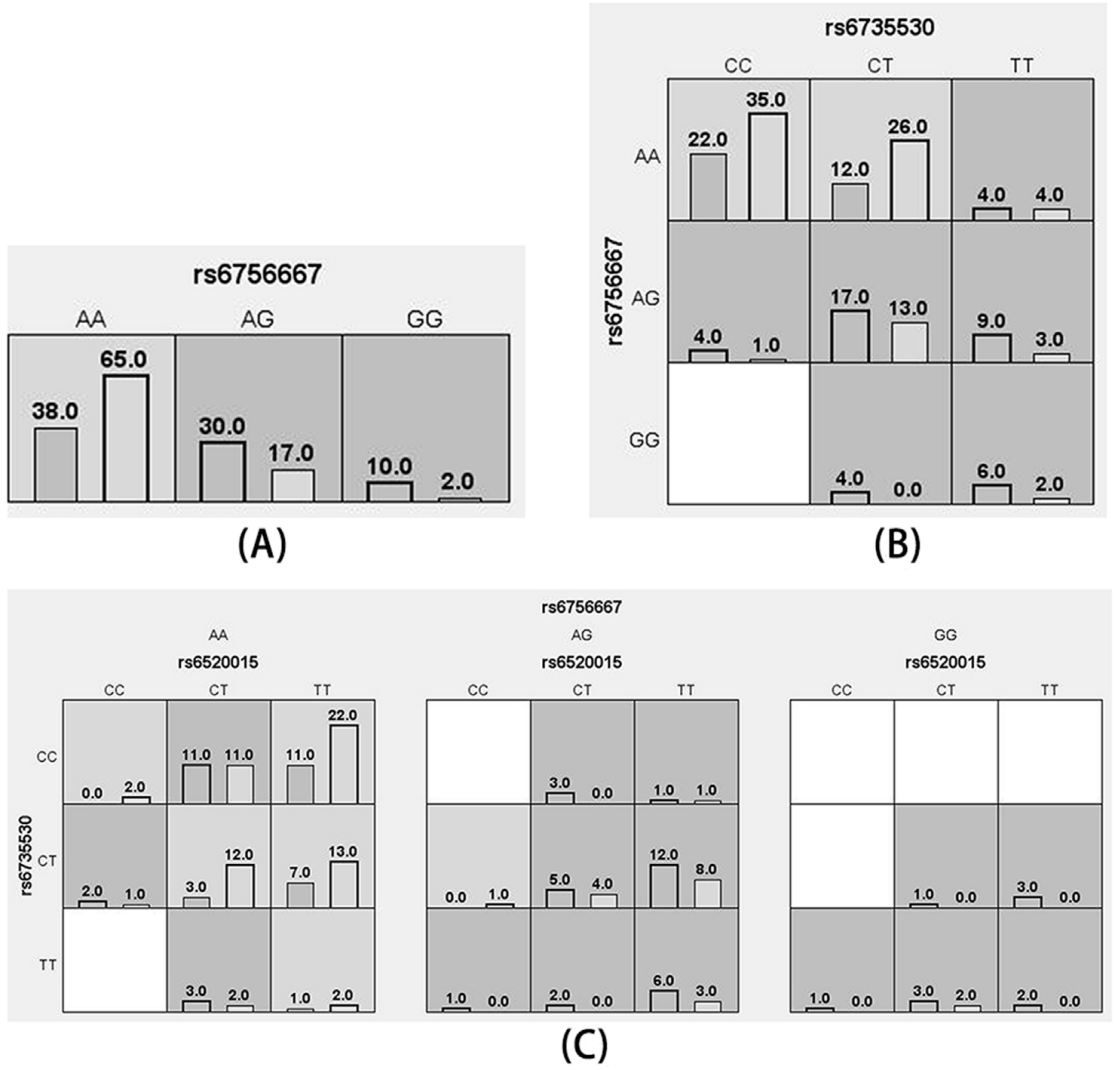
Cell diagram of the optimal model. **(A)**: rs6756667 model; **(B)**: rs6735530, rs6756667 model; **(C)**: rs6520015, rs6735530, rs6756667 model (black bars on the left side indicate the case group, black bars on the right side indicate the control group, one cell represents one interaction combination, light gray cells indicate that the ratio of the combination does not exceed the ratio threshold and is low-risk, dark gray indicates that the ratio threshold is exceeded and is high-risk, and white indicates that there is no data on the combination).

MDR analysis was used to analyze the association between interactions among the five SNP loci and high plateau erythrocytosis. Figure 3 shows a dendrogram of the interactions among the three loci obtained by MDR analysis. The blue line indicates that the SNPs have a redundancy effect in regulating the risk of HAPC, and the gold line represents the intermediate point between synergistic and redundancy effects. The closer the loci are, the stronger the interactions are. In this way, we investigated whether there were significant interactions between different loci to further explore the effect of SNP-SNP interactions on HAPC disease. The results showed that the cross-consistency was high in both the *EPAS1* rs6756667 model and the *PPARA* rs6520015, *EPAS1* rs6735530, and *EPAS1* rs6756667 model, which were 10 and 9, respectively, and the best prediction model was the *EPAS1* rs6756667 unit-point model (CVC: 10/10, test balance accuracy: 0.6433, P = 0.231), and in all three models the *P*-value was greater than 0.05, which was not statistically significant. It indicates that there is a strong interaction between gene loci, but there is no significant effect on the development of HAPC, and the results are shown in Table 6, Figures 3, 4.

**TABLE 6:** Multi-factor dimensionality reduction analysis.

Model	Training accuracy	Testing accuracy	*P* Value	CVC
rs6756667	0.6433	0.6433	0.231	10/10
rs6735530, rs6756667	0.6479	0.5893	0.4648	5/10
rs6520015, rs6735530, rs6756667	0.6708	0.5925	0.4568	9/10

CVC, CV consistency.

## 4 Discussion

More and more studies have suggested that genetic factors are one of the important factors contributing to the development of HAPC in Tibetan populations. *EPAS1* rs12619696, rs13419896, and rs4953354 were significantly associated with the risk of HAPC disease in Tibetan populations on the Tibetan Plateau (Xu et al., 2015b). HAPC is an adverse consequence of adaptation to the high altitude environment and is closely related to the low-pressure environment of plateau hypoxia. The increasing number of erythrocytes and hemoglobin concentration poses a great threat to the health status of the population at high altitude (Wang et al., 2019). Therefore, it is important to explore the risk of HAPC genetically to improve the knowledge and diagnosis of this disease.

One of the hotspots of previous studies on the correlation between hypoxia adaptation and genetic polymorphisms is the *EPAS1* gene, which encodes HIF-2α. Hypoxia-inducible factor (HIF) is a transcription factor that activates the adaptive hypoxia response at low levels of oxygen, and the two proteins, HIF-1 and HIF-2, consist of an unstable ɑ-chain and a stabilized β-chain (Jaśkiewicz et al., 2022; Yi et al., 2010); under normoxic conditions, the hydroxylation of HIF-1ɑ and HIF-2ɑ are post-translationally regulated by hydroxylation of specific proline residues within the oxygen-dependent degradation structural domain, which is carried out by prolyl hydrolase structural domain (PHD) proteins; hydroxylated HIF-α is recognized by von Hippel-Lindau (VHL) proteins, whose target HIFs are degraded via the ubiquitin-proteasomal pathway; in a hypoxic state, HIF-2ɑ hydroxylation is reduced, preventing the binding of HIF-2ɑ to VHL proteins, and the accumulated HIF-2ɑ continuously activates the HIF pathway, leading to a possible impact on the expression of downstream genes (Pamenter et al., 2020; Semenza, 2012; Sergi, 2019). Beall et al. found that *EPAS1* variants downregulate its transcript levels, while its encoded transcription factor HIF-2α stimulates erythropoiesis, resulting in an increase in blood Hb concentration (Beall et al., 2010). Gruber et al. find that postnatal *EPAS1* deficiency in mice causes anemia (Gruber et al., 2007). Tan et al. found that the *EPAS1* G536W missense mutation causes erythrocytosis (Tan et al., 2013). In this study, we found that the rs6735530, rs6756667, rs7583392, and rs12467821 polymorphisms of the *EPAS1* gene were significantly associated with HAPC in the Chinese Tibetan population by analysis. Among them, allele “G” in *EPAS1* rs6756667 and allele “T” in rs12467821 were both significantly associated with increased risk of HAPC, and the analysis of codominant, dominant, recessive and overdominant models for rs6756667, rs12467821 showed an increased risk of HAPC. The allele “T” in *EPAS1* rs6735530 is significantly associated with an increased risk of HAPC, and its codominant and recessive model analyses show an increased risk of HAPC. The allele “A” in *EPAS1* rs7583392 is significantly associated with an increased risk of HAPC, and its codominant, dominant, and recessive models also show an increased risk of HAPC.

Under hypoxia, PPARα encoded by the *PPARA* gene enhances the regulation of anaerobic glycolysis and lactate accumulation in the venous blood, thereby increasing oxygen utilization and obtaining sufficient ATP for the organism (Kinota et al., 2023). In Tibetans, the expression of the putative favorable *PPARA* haplotype and PPARα was significantly and positively correlated with fatty acid oxidative capacity (FAO), and reduced expression of PPARα could enhance the body’s efficiency in utilizing oxygen, suggesting that *PPARA* is associated with hypoxic metabolic adaptation (Murray et al., 2018; Horscroft et al., 2017). We found no correlation between *PPARA* rs6520015 and the risk of HAPC in the Chinese Tibetan population. This is consistent with the results of previous studies. It has been reported that HIF can regulate *PPARA* by repressing its promoter during hypoxia, which reduces hemoglobin concentration in Tibetan population (Bhandari and Cavalleri, 2019; Bai et al., 2012).

In addition, the results of haplotype testing in this study showed that the TCAGC haplotype at the rs6520015, rs6735530, rs6756667, rs7583392, and rs12467821 loci decreases the risk of HAPC in Tibetans, and the TTGAT haplotype increases the risk of HAPC in Tibetans. And rs7583392 is in complete linkage disequilibrium with rs12467821. It indicates that rs7583392 and rs12467821 are highly interlinked, and the chance of their alleles appearing together on the same chromosome is higher than the frequency of random appearance. We also predicted the interactions between these SNPs by MDR analysis and found that there was no effect on the development of HAPC in the *EPAS1* rs6756667 model and the *PPARA* rs6520015, *EPAS1* rs6735530, *EPAS1* rs6756667 model, although there was a strong interaction. This may have occurred because the MDR analysis could not adjust for covariates; and it could only analyze dichotomous phenotypes. It can only find interactions but does not identify the main effectors among the many factors. When we study fewer factors, there are limitations in using MDR analysis to accurately detect the effect of interactions on disease due to reduced dimensionality.

In this experiment, there are still some limitations. First, the sample size was small and limited to Tibetan patients in the Lhasa region of Tibet, so it is not possible to extrapolate the significance of the findings to other populations, and therefore the practical significance of the genetic polymorphisms needs to be investigated with larger sample sizes and different populations; second, we studied fewer loci, and in the future, we should investigate the correlation between other polymorphic loci in the *EPAS1* and *PPARA* gene and the prevalence of HAPC.

## 5 Conclusion

In the Chinese Tibetan population, *EPAS1* rs6735530, rs6756667, rs7583392, and rs12467821 polymorphisms were associated with the risk of developing HAPC, while *PPARA* rs6520015 was not significantly associated with HAPC.

## Data Availability

The original contributions presented in the study are included in the article/Supplementary Material, further inquiries can be directed to the corresponding authors.
